# Increases in both temperature means and extremes likely facilitate invasive herbivore outbreaks

**DOI:** 10.1038/srep15715

**Published:** 2015-10-27

**Authors:** Rui-Ting Ju, Hai-Yan Zhu, Lei Gao, Xu-Hui Zhou, Bo Li

**Affiliations:** 1 Ministry of Education Key Laboratory for Biodiversity Science and Ecological Engineering, Fudan University, 2005 Songhu Road, Shanghai 200438, China; 2 Department of Plant Protection, Shanghai Landscape Gardening Research Institute, 899 Longwu Road, Shanghai, 200232, China; 3 School of Life Sciences, Anqing Teachers College, 128 South Linghu Road, Anqing, Anhui 246011, China

## Abstract

Although increases in mean temperature (MT) and extreme high temperature (EHT) can greatly affect population dynamics of insects under global warming, how concurrent changes in both MT and EHT affect invasive species is largely unknown. We used four thermal regimes to simulate the increases in summer temperature and compared their effects on the life-history traits of three geographical populations (Chongqing, Wuhan and Shanghai) of an invasive insect, *Corythucha ciliata*, in China. The four thermal regimes were control (i.e., natural or ambient), an increase in MT (IMT), an increase in EHT, and a combination of IMT + EHT. We found that the three warming regimes significantly increased the developmental rate but did not affect the survival, sex ratio, longevity, or fecundity of *C. ciliata*. Consequently, the intrinsic rate of natural increase (*r*_m_) was enhanced and the number of days required for population doubling (*t*) was reduced by the warming regimes. The demographic parameters did not significantly differ among the three populations. These results indicate that population size of *C. ciliata* may be enhanced by increases in both temperature means and extremes. The increases in summer temperature associated with climate change, therefore, would likely facilitate population outbreaks of some thermophilic invasive insects.

Climate change and biological invasions are two important drivers affecting native ecosystems and regional economies^1^,^2^,^3^,^4^. Given that biological invasions may be facilitated by environmental change^5^, the responses of invasive species to climate change have been receiving increasing attention in global change ecology and invasion biology^6^. In particular, it has been a focal issue about how the temporal and spatial dynamics of invading populations respond to climate change in recent years^7^. To address this issue, we must first understand how climate change affects the life-history traits (e.g., development, survival, reproduction, and etc.) of invasive species because it is a decisive factor for their colonization in the introduced regions.

Under climate change, global warming does not involve an increase in mean temperature (MT) only but also in the frequency of extreme high-temperature (EHT) events. The latest IPCC report on climate change indicates that global MT has risen by 0.85 °C since 1880 and is projected to increase another 1.5 to 4.5 °C by the end of this century^8^. Such an increase in MT is likely to be accompanied by more frequent and more severe EHT events because even a small rise in MT can dramatically increase heat waves^9^. Although changes in MT and EHTs can strongly affect the fitness of organisms, previous studies have mainly concentrated on their individual effects on native species^10^; the impacts of concurrent changes in MT and EHT on invasive species have rarely been studied^11^.

As ectotherms, insects are sensitive to changes in MT as well as to temperature fluctuations. Previous studies have shown that global warming is likely to have substantial effects on insects^12^,^13^. Increased MT, for example, enhances insect overwintering survival^14^, growth, and fecundity^15^, which may increase the number of generations per year^16^ and change insect distribution^17^ and phenology^18^. On the other hand, insects are often more affected by EHTs than by MTs^9^. If the frequency and severity of EHT events continue to increase, the thermal limits of many insect species may be exceeded in the warming future^19^, which will lead to decline in insect populations^20^,^21^. Many invasive insects, however, possess great thermal plasticity that may enable them to adapt to severe EHT events^22^,^23^. Such species may benefit from the higher MTs and the more EHTs^24^. As noted earlier, however, there are few studies available concerning the combined effects of changes in MT and EHT on these insect species.

In this study, we investigated how changes in MT, EHT, and their combination affect the invasive lace bug, *Corythucha ciliata* (Hemiptera: Tingidae). Although *C. ciliata* is native to temperate North America, it now occurs throughout Europe, Australia, and Asia. This lace bug feeds on and damages trees in the genus *Platanus* in the invaded regions, resulting in stunted growth and sometimes in tree deaths^25^. In China, *C. ciliata* was first found in Hunan Province in 2002 and has since spread to 11 other eastern and northern provinces between latitudes 26 °N and 37 °N^26^,^27^. The most severe damage caused by this species has been in the Yangtze Basin, where it has become a common pest that feeds on *Platanus* trees mainly in summer (June–August)^28^,^29^. Over the last decade, we found that *C. ciliata* abundance in summer had generally increased in the Yangtze Basin and had been higher in hotter than in cooler years.

Considering that MTs and the frequency and intensity of EHT events may continuously increase in the Yangtze Basin due to global warming^30^, we hypothesized that cyclical heat activations may benefit population outbreaks of *C. ciliata* in summer. To test this hypothesis, we conducted a controlled experiment to compare the effects of four temperature regimes on the population fitness of the insect. Among these regimes, one simulated ambient temperature and is abbreviated as NAT (natural) (i.e., control); the second simulated an increase in MT and is abbreviated as IMT; the third simulated the enhanced high temperature and is abbreviated as EHT; and the fourth combined IMT and EHT and is abbreviated as MHT. The insects were collected from three geographical sites in the Yangtze Basin, including Shanghai (representing the lower basin), Wuhan (representing the middle basin), and Chongqing (representing the upper basin). We attempted to answer the following two questions: (i) Do different warming patterns during diurnal cycles in summer affect the development, reproduction, survival, and demographic parameters of the three geographic populations of *C. ciliata*? and (ii) How do these findings translate into population prediction under global warming?

## Results

### Development, sex ratio, and survival

**Figure 1:**
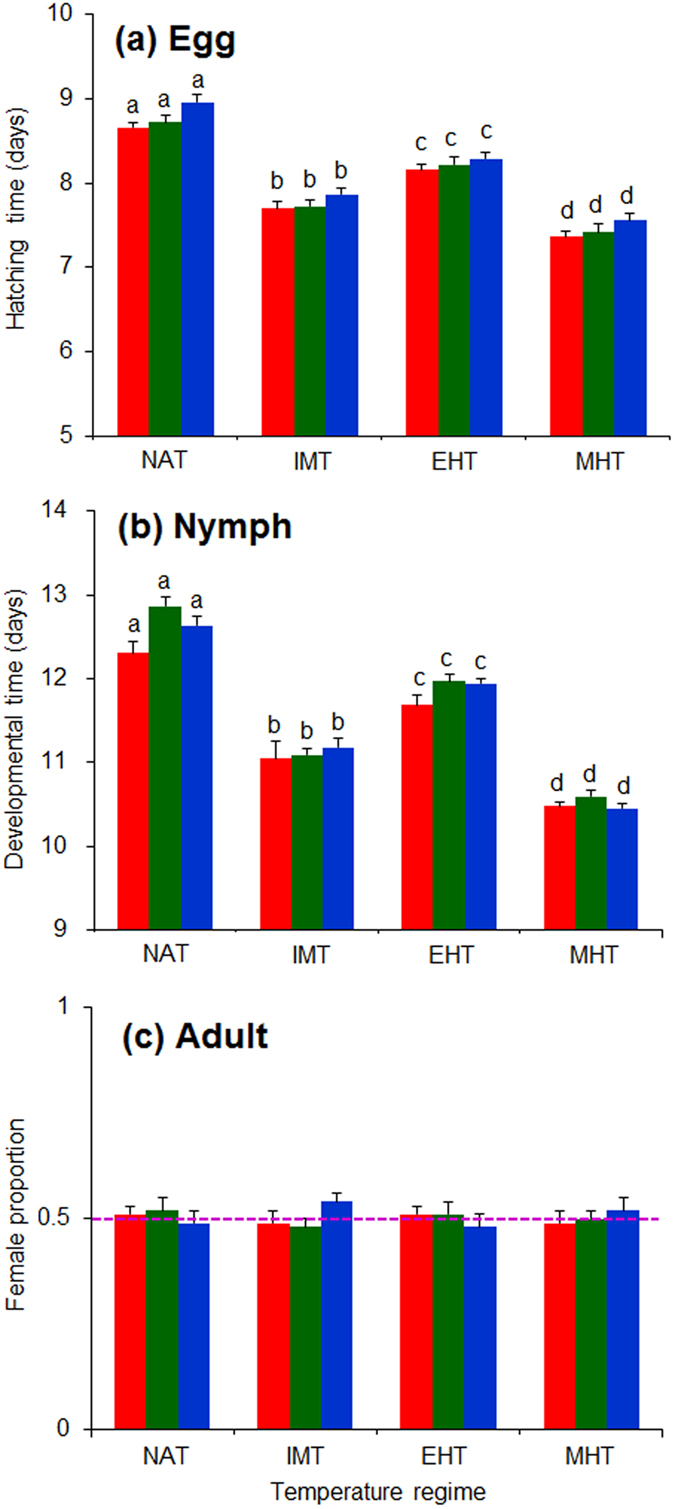
Hatching time of eggs (a), developmental time of nymphs (b), and the proportion of adults represented by females (c) of three populations of *C. ciliata* as affected by four temperature regimes. Red, green and blue columns represent the Shanghai, Wuhan, and Chongqing population, respectively. The temperature regimes are described in Fig. 6. Values are means + SE. Means in panels with different letters are significantly different (Tukey’s test, *P* < 0.001, one-way ANOVA) for the hatching/developmental time. ANOVA statistics are provided in Table 1.

**Figure 2:**
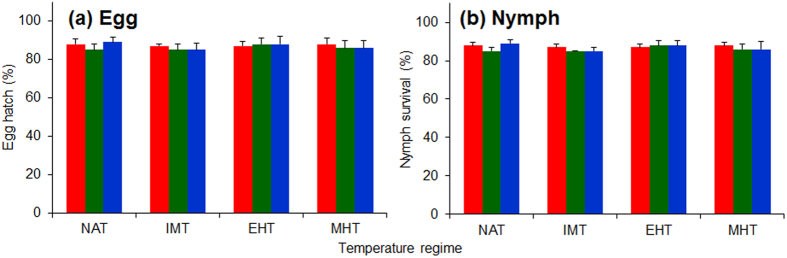
Egg hatch (a) and nymph survival (b) of three populations of *C. ciliata* as affected by four temperature regimes. Red, green and blue columns represent the Shanghai, Wuhan, and Chongqing population, respectively. The temperature regimes are described in Fig. 6. Values are means + SE. ANOVA statistics are provided in Table 1.

The developmental times for the eggs and nymphs of *C. ciliata* were significantly shorter in the IMT, EHT, and MHT regimes than in the NAT regime (control, Fig. 1a,b and Table 1). Development was the fastest with MHT, followed by IMT, and then EHT (Fig. 1a,b). Population provenance significantly affected egg hatching time but not developmental time of nymphs (Table 1). Developmental times of eggs and nymphs were not influenced by the interaction between population provenance and temperature regime (Table 1). Females represented about one-half of the adults, regardless of population provenance or temperature regime (Fig. 1c and Table 1). Survival rates of eggs (>90%; Fig. 2a) and of nymphs (>85%; Fig. 2b) were not affected by population provenance, temperature regime, or their interaction (Table 1).

### Reproduction and longevity

**Figure 3:**
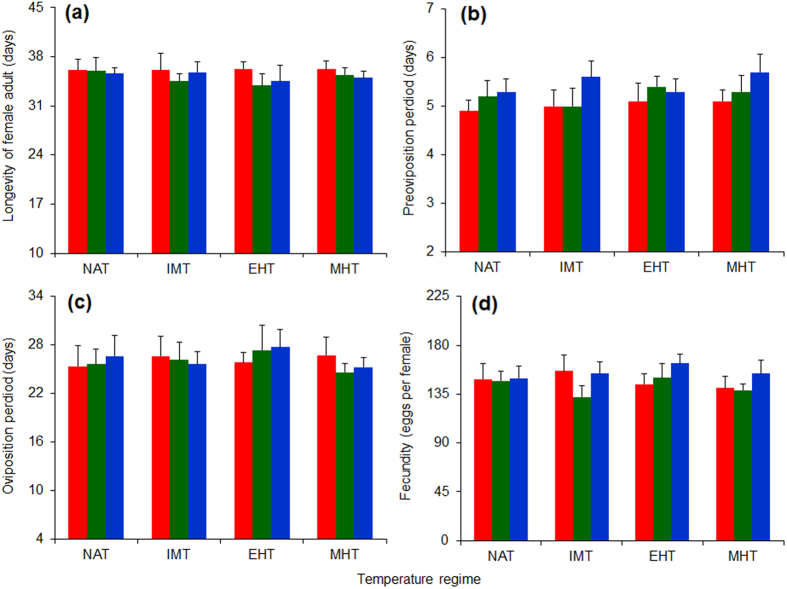
Longevity (a), preoviposition period (b), oviposition period (c), and fecundity (d) of female adults of three populations of *C. ciliata* as affected by four temperature regimes. Red, green and blue columns represent the Shanghai, Wuhan, and Chongqing population, respectively. The temperature regimes are described in Fig. 6. Values are means + SE. ANOVA statistics are provided in Table 1.

Female longevity (Fig. 3a), preoviposition period (Fig. 3b), oviposition period (Fig. 3c), and fecundity (Fig. 3d) for the adults of *C. ciliata* were not significantly affected by temperature regime or population provenance (Table 1). Also, these life-history parameters were unaffected by the interaction of population provenance and temperature regime (Table 1).

### Demographic parameters

**Figure 4:**
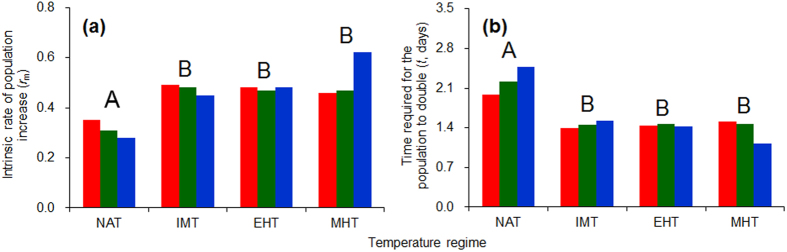
The intrinsic rate of population increase (*r*_m_) (a) and the time required for population doubling (*t*) (b) for three populations of *C. ciliata* as affected by four temperature regimes. Red, green and blue columns represent the Shanghai, Wuhan, and Chongqing population, respectively. The temperature regimes are described in Fig. 6. Different capital letters indicate significant differences among the warming regimes (*P* < 0.05). ANOVA statistics are provided in Table 1.

The intrinsic rate of population increase (*r*_m_) for *C. ciliata* was higher in IMT, EHT, and MHT than in NAT (Table 1 and Fig. 4a). Consequently, the time required for population doubling (*t*) was significantly shorter in the warming regimes than that in the natural regime (Table 1 and Fig. 4b). The demographic parameters were not significantly affected by population provenance (Table 1) and were also similar among IMT, EHT, and MHT regimes (Fig. 4a,b).

## Discussion

As indicated in a recent review^31^, many non-native insect species may be favored by climate change because of their abilities to rapidly acquire resources, grow, and reproduce in disturbed areas. The effects of global warming on insects may differ depending on the thermal sensitivities and specific requirements of particular species, as well as on the warming modes and intensities that act on them^32^. Although increased MTs coupled with more frequent EHT events are expected to greatly influence the survival and spread of many insect species^33^, few studies have considered the effects of concurrent changes in both MT and EHT. The current study showed that either or both of the increases in MT and EHT might increase the abundance of the invasive lace bug, *C. ciliata*, through maintaining fecundity and accelerating development. Our results are consistent with the view that some invasive insects, if they have great ecological plasticity, may benefit from environmental changes^22^,^23^.

Our previous study found that neither eggs nor nymphs of *C. ciliata* developed at constant temperatures ≥36 °C^34^. In the present study, however, the eggs and nymphs developed well with a diurnal cycle that included a treatment of 38 °C for 2 h (the EHT regime), indicating that the insects can develop at a high temperature extreme under fluctuating conditions. Compared to developmental rates in the natural regime, developmental rates of *C. ciliata* were most enhanced by the MHT regime (mean temperature = 30.1 °C), followed by the IMT regime (mean temperature = 29.8 °C) and then by the EHT regime (mean temperature = 28.3 °C). This suggests that developmental rates of *C. ciliata* under different fluctuating conditions might still depend on mean temperatures, which was different from a previous work having differential effects on the development of a mosquito (*Anopheles stephensi*)^32^ under different temperature fluctuations with the same means. When the effects of the fluctuating temperatures with a mean temperature of 30.1 °C in the current study are compared with those obtained with a constant 30 °C in our previous study^34^, however, developmental times for eggs and nymphs were 1.5–2.2 days shorter with the fluctuating temperatures than with the constant temperature. This difference might have resulted from the “rate summation effect”, i.e., fluctuations above the mean have a greater effect on developmental rates than similar fluctuations below the mean^35^.

In addition to the effect on insect development, global warming can also affect insect survival^19^. Survival of eggs and nymphs of *C. ciliata* was as high with fluctuating temperatures and even with 2 h at 38 °C in the daily cycle of the current study as with a constant temperature of 30 °C in our previous study^34^. This suggests that the fluctuating temperatures used in the current study did not exceed the upper thermal limit of *C. ciliata*. Even if *C. ciliata* regularly experienced thermal stress at 38 °C in the current study, the insects might have gained thermal plasticity through rapid heat hardening^28^. The maximum temperature described in the experiment occurred between 14:00–16:00 and was preceded by moderate and gradually increasing temperatures in the morning. The gradual increase may induce rapid heat hardening. Under natural condition, rapid heat hardening often helps insects tolerate higher temperatures under climate change^22^,^36^,^37^.

Increases in temporal or seasonal temperature *per se* have been shown to alter fecundity and sex ratios in many insect species, such as those from Diptera, Lepidoptera, Coleoptera, Hemiptera and Hymenoptera^21^,^38^,^39^,^40^,^41^,^42^,^43^,^44^,^45^,^46^. In this study, however, none of the increased-temperature regimes affected the sex ratio, longevity, oviposition, or fecundity of *C. ciliata*. These results indicate that a hotter summer is unlikely to have negative effects on the sex ratio and reproduction of *C. ciliata* under the future global warming.

The ecology and physiology of organisms are often linked to large-scale geographical patterns of environmental conditions^47^,^48^. Although the three populations of *C. ciliata* investigated in this study were collected from three distant locations of the Yangtze Basin with similar latitude (29.6–31.1 °N), the local climate differs among these locations because of differences in elevation and terrain^49^,^50^. The insects, however, responded similarly to the temperature regimes, indicating that the thermal adaptation of *C. ciliata* is similar among populations in the Yangtze Basin. We therefore suggest that *C. ciliata* might have evolved the ability to adapt to a wide range of thermal conditions before it spread into subtropical China, which may partially explain its success as an invasive insect.

**Figure 5:**
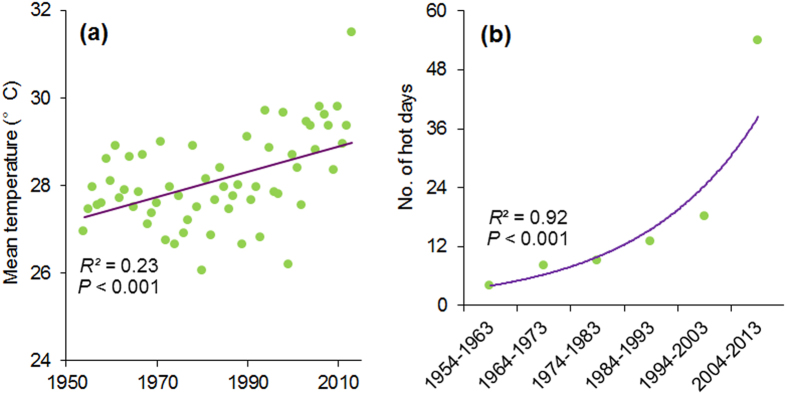
Mean temperatures (a) and number of days in which the daily maximum temperatures were ≥37 °C (b) in July-August at Shanghai from 1954 to 2013. Data were obtained from the China Meteorological Data Sharing Service System (http://cdc.cma.gov.cn). The weather stations providing these data are located in Longhua, Shanghai (31.10 °N, 121.26 °E).

Based on the effects of global warming on insects^12^,^31^,^51^, predicting an insect’s population dynamics requires an understanding of how the insect responds to thermal conditions. We previously found that *C. ciliata* could survive at heat-shock temperatures as high as 40 °C for 2 h before they were transferred to a constant temperature of 26 °C^29^. In the present study, we further found that increasing the current diurnal temperature cycle of summer by 2 °C in the IMT regime, increasing the peak temperature to 38 °C for 2 h in the EHT regime, or combining these two regimes enhanced the population growth of *C. ciliata*. Given current temperatures and climate change in the Yangtze Basin^30^, for example in Shanghai, MTs in summer have steadily increased over the last 60 years (Fig. 5a), while the number of hot days on which the daily maximum temperature was greater than 37 °C was 12.5-times greater for the most recent 10-year period than for the 10-year period of 1954–1963 (Fig. 5b). Hot summers in recent years, therefore, might have contributed to population outbreaks of *C. ciliata* in the Yangtze Basin, where severer outbreaks of the invasive pest will likely occur in the future due to more hot-summers caused by climate warming.

Given climate change in general and global warming in particular, the spread and establishment of an alien insect, such as *C. ciliata*, may largely depend on its thermo-adaptability^4^. Understanding the responses of invasive insects to thermal conditions, therefore, is crucial to predict the shifts in their potential biogeographic range in response to climate change. Many previous studies have shown that, because of global warming, many insect species are advancing toward higher latitudes and higher elevations^52^,^53^, and tend to retreat from lower latitudes and lower elevations^19^,^54^,^55^. However, the data in this study indicate that *C. ciliata* is likely to spread into lower latitudes in China where host plants are widely planted, because high temperatures may benefit the invader. An increase in summer temperatures, therefore, probably facilitates disperses and range expansions of some introduced, invasive thermophilic insects. Overall, the role of increasing MTs and EHTs on the establishment and spread of such insect species should be considered in the context of climate change and invasion biology. Such information will be needed to conduct more accurate risk assessments and to improve management strategies. Further studies are needed to cover wider temperature regimes and fluctuations than the conditions considered in the present study to simulate more complex conditions of climate change in the future. Additionally, follow-up experiments are also needed to compare the different responses to similar thermal regimes between invasive and non-invasive species, which will substantially enhance the persuasion on the positive effects of temperature variability and increases on invasive species.

## Materials and Methods

### Collection and maintenance of insects

Laboratory colonies of *C. ciliata* were established in June 2014 from adults collected from London plane trees (*Platanus* × *acerifolia*) at Shanghai (31.1 °N, 121.3 °E, 5 m a.s.l.), Wuhan (30.5 °N, 114.4 °E, 23 m a.s.l.), and Chongqing (29.6 °N, 106.5 °E, 259 m a.s.l.) in the Yangtze Basin, China. However, there was no biological replication of geographical area due to space limitation. These populations (one per geographical area) were separately reared on fresh leaves of *P.* × *acerifolia* in closed Petri dishes (15-cm diameter; 20 individuals per dish) in Shanghai according to their provenances. More than 200 adults were used for the starting culture of each geographical population. The fresh leaves were replaced daily to ensure enough food supply. The stock cultures were uniformly maintained in the laboratory at 26 ± 0.5 °C with a relative humidity (RH) of 80 ± 5% and a 14 h:10 h (L:D) photoperiod, regardless of population provenances. Newly oviposited eggs by the adults of the *F*_2_ generation were used for the starting experiment.

### Temperature regimes

**Figure 6:**
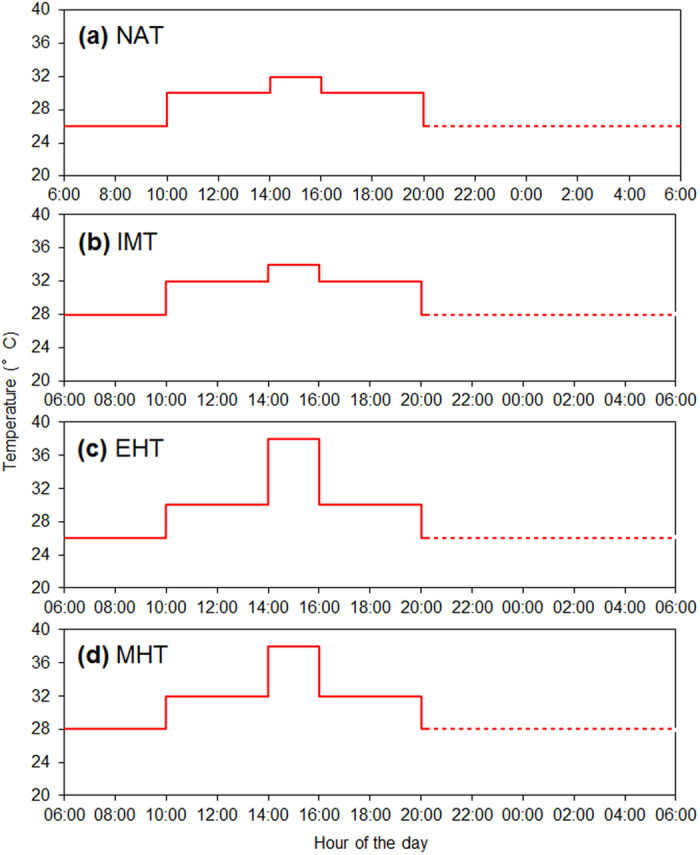
The four temperature regimes in the experiment. NAT (natural regime), temperatures ranging from 26 to 32 °C in a diurnal cycle that simulated the natural cycle in summer (**a**); IMT (an increase in mean temperature regime), identical to NAT but 2 °C warmer throughout the diurnal cycle (**b**); EHT (extreme high temperature regime), identical to NAT but with a peak temperature of 38 °C at 14:00–16:00 (**c**); MHT (IMT + EHT regime), a combination of IMT and EHT (**d**). Full and broken red lines indicate the light and dark parts, respectively, of the photoperiod.

To compare the effects of different thermal conditions on the fitness of *C. ciliata* in summer, we conducted an experiment with four temperature regimes. One regime, designated “natural” (i.e., control) and abbreviated as NAT, involved diurnal temperatures that increased from 26 °C (low) to 32 °C (high) in two steps and then returned to the low temperature in two steps (Fig. 6a). The natural regime was based on the ambient temperature fluctuations in summer in the Yangtze Basin^49^. The second regime was designated “an increase in MT” and was abbreviated as IMT; the temperature was 2 °C higher than NAT (Fig. 6b). A 2 °C warming was referred to as the critical mean increase based on IPCC^8^. The third regime was designated “extreme high temperature” and abbreviated as EHT; the temperature was the same as NAT except that it increased to 38 °C rather than to 32 °C from 14:00 to 16:00 (Fig. 6c). The EHT regime was selected based on extreme high-temperature events occurring in the Yangtze Basin over the last 10 years^50^,^56^. The fourth regime combined IMT and EHT and was abbreviated as MHT (Fig. 6d). The diurnal cycles of each temperature regime were maintained in a climatic incubator (MIR 350H, Sanyo Electric Co. Ltd., Osaka, Japan). Based on our previous studies^28^,^29^,^34^, the photoperiod was 14 h:10 h (L:D), and the RH was 80 ± 5%.

### Development and survival of immature insects

For each *C. ciliata* population (Shanghai, Wuhan, and Chongqing), one *P.* × *acerifolia* leaf with at least 30 *C. ciliata* eggs was placed in a closed Petri dish (15-cm diameter) with a piece of wet filter paper. The eggs had been deposited on the leaf <24 h earlier. The Petri dishes were placed in the four incubators (one incubator per temperature regime). The wet filter paper was replaced daily. The leaves were examined daily, and the number of eggs that hatched was recorded. Egg survival and the time required for eggs to hatch were determined for each regime. Each combination of population and temperature regime was represented by five replicate dishes, with >30 eggs per replicate.

After the eggs hatched, newly emerged nymphs (i.e., nymphs of the same age in days) were placed in new closed Petri dishes (15-cm diameter; 20 nymphs per dish) with a *P.* × *acerifolia* leaf in each dish. The nymphs, which were subjected to the same temperature regime as the eggs from which they hatched, were examined daily to measure survival and the time required for development into adults. Leaves were replaced daily. After eclosion of all adults, the sex ratio in each dish was determined. Each combination of population and temperature regime was represented by five replicate dishes, with 20 nymphs per replicate.

### Fecundity, oviposition, and longevity of adults

When adults emerged from the nymphs that had been maintained under the four temperature regimes, they were paired (one male and one female), and each pair was placed on a fresh *P.* × *acerifolia* leaf in a Petri dish (15-cm diameter). The pairs were subjected to the same temperature regime as the nymphs from which they developed. The leaf acted as a food source and oviposition site. Dishes were examined daily for oviposition. When oviposition occurred, leaves with attached eggs were removed, and new leaves were supplied until the female died. The eggs deposited on leaves that had been removed from the dishes were examined daily with a binocular stereoscope (MZ 16A, Leica Microsystems Ltd., Wetzlar, Germany). As described in our previous reports^29^,^34^, the following data were collected: length of the preoviposition period, length of the oviposition period, female longevity, and fecundity. At least 10 pairs of adults (one pair per replicate) were used for each combination of population and temperature regime.

### Data analysis

Two-way analysis of variance (ANOVA) was used to test the effects of *C. ciliata* population provenance, temperature regime, and their interaction on the life-history traits and demographic rates of *C. ciliata*. The effects of temperature regimes on life-history traits of *C. ciliata* from the same population were analyzed by one-way ANOVA. Results are presented as means and SE, and the means were compared with Tukey’s test. Before analysis, all data were checked for normality and homoscedasticity. Survival and female-proportion data were log-transformed before statistical analysis. The analyses were performed with the statistical package SPSS NLN, 15.0 (SPSS Inc., Chicago, USA).

The intrinsic rate of increase (*r*_*m*_) and the number of days required for population doubling (*t*) were calculated from the net reproductive rate (*R*_*0*_) and the mean generation time (*T*) according to the following equations^57^,^58^:


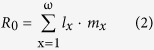



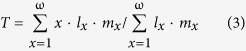



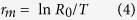



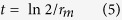


where *x* is the age in days of the insect from egg hatching to adult dying, *l*_*x*_ is the daily survival rate (expressed as a percentage) of the immature and adult (female) stages, and *m*_*x*_ is the age-specific oviposition rate (expressed as the number of eggs).

## Additional Information

**How to cite this article**: Ju, R.-T. *et al.* Increases in both temperature means and extremes likely facilitate invasive herbivore outbreaks. *Sci. Rep.* **5**, 15715; doi: 10.1038/srep15715 (2015).

## Figures and Tables

**Table 1:** Summary of two-way ANOVAs on the effects of population provenance^a^ (P), temperature regime (T), and their interaction on life-history traits and demographic parameters of *C. ciliata*.

Developmental stage	Trait/parameter	Source of variance	*df*	*F*	*P*
Egg	Hatching time	P	2, 1024	6.63	<0.001
		T	3, 1024	265.71	<0.001
		P × T	6, 1024	0.28	0.743
	Survival	P	2, 59	0.18	0.836
		T	3, 59	0.35	0.789
		P × T	6, 59	0.12	0.994
Nymph	Developmental time	P	2, 968	2.47	0.084
		T	3, 968	344.33	<0.001
		P × T	6, 968	0.78	0.458
	Survival	P	2, 59	0.27	0.766
		T	3, 59	0.23	0.878
		P × T	6, 59	0.17	0.984
Adult	Female proportion	P	2, 59	0.37	0.781
		T	3, 59	0.21	0.924
		P × T	6, 59	0.19	0.897
	Female longevity	P	2, 119	16.52	0.493
		T	3, 119	5.12	0.882
		P × T	6, 119	3.58	0.987
	Preoviposition period	P	2, 119	2.12	0.124
		T	3, 119	0.31	0.818
		P × T	6, 119	0.27	0.951
	Oviposition period	P	2, 119	0.03	0.968
		T	3, 119	0.26	0.855
		P × T	6, 119	0.21	0.973
	Fecundity	P	2, 119	1.40	0.252
		T	3, 119	0.26	0.855
		P × T	6, 119	0.45	0.843
Generation	*r*_m_^b^	P	2, 11	0.21	0.819
		T	3, 11	7.99	0.016
	*t*^c^	P	2, 11	0.27	0.776
		T	3, 11	13.20	0.005

^a^The experiment used three populations of *C. ciliata* that originated in different geographical areas of the Yangtze Basin.

^b^The intrinsic rate of population increase.

^c^The time required for population doubling.
